# Reduction in Serum High-Sensitivity C-Reactive Protein Favors Kidney Outcomes in Patients with Impaired Fasting Glucose or Diabetes

**DOI:** 10.1155/2020/2720905

**Published:** 2020-06-07

**Authors:** Lili Liu, Bixia Gao, Jinwei Wang, Chao Yang, Shouling Wu, Yuntao Wu, Shuohua Chen, Qiuyun Li, Huifen Zhang, Guodong Wang, Min Chen, Ming-hui Zhao, Luxia Zhang

**Affiliations:** ^1^Renal Division, Department of Medicine, Peking University First Hospital, Peking University Institute of Nephrology, Key Laboratory of Renal Disease, Ministry of Health of China, Key Laboratory of Chronic Kidney Disease Prevention and Treatment, Ministry of Education, Beijing 100034, China; ^2^Department of Cardiology, Kailuan General Hospital, Tangshan 063001, China; ^3^Department of Health Care Center, Kailuan General Hospital, Tangshan 063001, China; ^4^Department of Endocrinology, Kailuan General Hospital, Tangshan 063001, China; ^5^Department of Laboratory, Kailuan General Hospital, Tangshan 063001, China; ^6^Peking-Tsinghua Center for Life Sciences, Beijing 100034, China; ^7^National Institute of Health Data Science at Peking University, Beijing 100191, China

## Abstract

**Objective:**

We aimed to evaluate whether the reduction in serum high-sensitivity C-reactive protein (hs-CRP) favors kidney outcomes.

**Methods:**

This study was a subanalysis including patients with impaired fasting glucose or diabetes of the Kailuan cohort study. The predictor was based on two consecutive visits of hs-CRP levels in 2006 and 2008. A total of 3924 patients with hs-CRP ≥ 3 mg/L in 2006 were divided into two groups according to whether the levels of hs-CRP were reduced in 2008: Group 1: no reduction: hs-CRP ≥ 3 mg/L in 2008; Group 2: reduction: hs-CRP < 3 mg/L in 2008. Kidney outcomes include kidney function decline and development and progression of proteinuria and were followed up until the end of 2015.

**Results:**

There were 3905, 2049, and 493 patients included into our analysis for the outcomes of kidney function decline and the development and progression of proteinuria, respectively. A total of 398, 297, and 47 events occurred after 5 years of follow-up, respectively. Cox regression revealed that patients with reduction in hs-CRP have lower risk of kidney function decline (HR 0.71, 95% CI 0.57-0.89, and *P* = 0.002) and development of proteinuria (0.77, 0.61-0.99, and *P* = 0.038) after controlling for potential confounders as compared to those with no reduction in hs-CRP levels.

**Conclusions:**

Reduction in serum hs-CRP levels favors kidney outcomes in patients with impaired fasting glucose or diabetes.

## 1. Introduction

Chronic kidney disease (CKD) has become a common public health problem all over the world, with diabetes as the main etiology [1]. Even people with prediabetes suffer an increased risk for the onset and progression of CKD [2]. In 2019, China has the highest number of diabetes in the world, with 116.4 million adults being affected and is expected to reach 147.2 million by 2045[3]. Epidemiologic studies have shown that the prevalence of CKD is 10.8% among general population and 21.3% among diabetes in China [4, 5].

Inflammation plays a relevant role in the development of CKD. The most commonly used biomarker of inflammation is high-sensitivity C-reactive protein (hs-CRP), which could predict all-cause and cardiovascular mortality [6]. Previous studies have reported that the elevated levels of hs-CRP could increase the risk of CKD among people with or without diabetes [7–9], while the evidence was not consistent [10, 11]. A study including 1301 participants with type 2 diabetes demonstrated a predictive value of hs-CRP for diabetic kidney disease after 7.5 years of follow-up [9]. However, in another study based on 1441 patients with type 1 diabetes, no significant effect of hs-CRP was observed on the change of the urinary albumin excretion rate [11]. To the best of our knowledge, among patients with high levels of hs-CRP, whether the reduction in hs-CRP levels will favor kidney outcomes has not been evaluated.

Therefore, we conducted this study aiming to evaluate whether the reduction in serum hs-CRP levels favors kidney outcomes in patients with impaired fasting glucose (IFG) or diabetes.

## 2. Materials and Methods

### 2.1. Study Population

Our study was based on the healthcare database from the Kailuan cohort [12], which was started in June 2006. The Kailuan Group has recruited 101,510 employees (aged 18 to 98 years and 81,110 males) to participate in the physical examination every two years in 11 hospitals that are responsible for the healthcare of the community.

We have obtained data on 30,016 patients with IFG or diabetes. According to the American Diabetes Association criterion, IFG was defined by fasting blood glucose (FBG) ≥ 100 and < 126 mg/dL (i.e., ≥ 5.6 and < 7.0 mmol/L) [13], with no history of diabetes and not using hypoglycemic drugs. Diabetes was detected by the cut-off point of FBG ≥ 126 mg/dL (7.0 mmol/L), and/or self-reported diabetes, and/or using hypoglycemic drugs.

**Figure 1:**
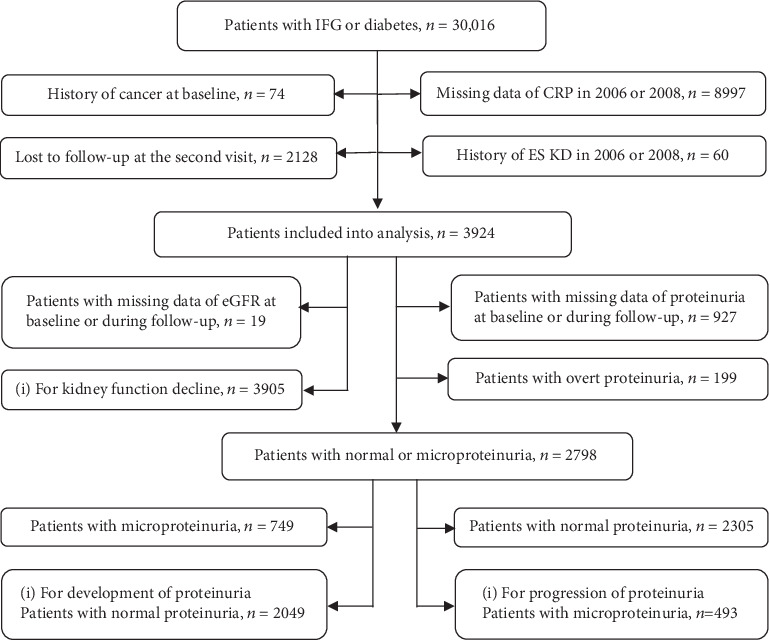
The detailed flow chart of study population.

In the current study, we selected 3924 patients with IFG or diabetes and with hs-CRP ≥ 3 mg/L at baseline, a frequently used cut-off point for identifying high-risk population [14]. The flow chart of study population is illustrated in Figure 1.

The Kailuan cohort study was approved by the Ethics Committee of Kailuan General Hospital. All participants provided informed consent in order to obtain their data for research purposes.

### 2.2. Data Collection

During the face-to-face survey, a questionnaire including the sociodemographic information, medical history, and living habits was accomplished by all participants. Height, weight, and waist circumference (WC) were measured in line with the protocol. Body mass index (BMI, kg/m^2^) was computed as weight/height^2^. Blood pressure (BP) was taken twice with a standardized mercury sphygmomanometer at a five-minute timespan after taking at least a five-minute break. If the difference between the two measurements was greater than 5 mmHg, one more BP was taken and the mean value of the three readings was adopted.

After an overnight fast for at least 8 hours, blood samples were drawn in the morning. Serum creatinine, FBG, and lipid profile were measured by utilizing a Hitachi 7600 autoanalyzer (Hitachi, Tokyo, Japan). The CKD Epidemiology Collaboration equation was performed for calculating the estimated glomerular filtration rate (eGFR) [15]. A clean midstream urine sample was gathered from each participant in the morning. All urine samples were analyzed by utilizing a urine analyzer (N-600, Dirui, Changchun, China) at the central laboratory of Kailuan Hospital. Proteinuria concentration was determined by the dry chemistry method with the test assay of H12-MA (Changchun Dirui Medical Technology Co., Ltd., Changchun, China). There are five levels of the semiquantitative dipstick: negative, trace, +, ++, or +++. The microproteinuria was considered urine dipstick reading “trace” or “+”, and overt proteinuria was considered urine dipstick reading “++” or “+++” [16].

### 2.3. Measurement of hs-CRP and Definition of Exposure

hs-CRP was tested by the immunoturbidimetry method (Kanto Chemical Co., Inc., Tokyo, Japan), and the lower bound of measurement was 0.1 mg/L. Precision was assessed by measuring hs-CRP concentration in two common serum samples twice a day, greater than or equal to a 2-hour interval for 20 days, and the within- and between-assay coefficients of variation of this method were 6.53% and 4.78%, respectively. The interday and overall coefficients of variation were 6.61% and 9.37%, respectively [17].

Patients with hs-CRP ≥ 3 mg/L at the first visit were divided into two groups according to whether the hs-CRP level was reduced or not at the second visit: Group 1: no reduction: hs-CRP ≥ 3 mg/L at the second visit; Group 2: reduction: hs-CRP < 3 mg/L at the second visit.

### 2.4. Kidney Outcomes

Kidney outcomes include the following: (1) kidney function decline, defined as eGFR decline ≥ 30% in two years [18], or doubling of serum creatinine concentration, or the onset of ESKD (eGFR < 15 mL/min/1.73 m^2^); (2) development of proteinuria, defined as the conversion from negative to micro- or overt proteinuria; and (3) progression of proteinuria, defined as the conversion from micro- to overt proteinuria.

### 2.5. Other Potential Covariates

Hypertension was defined as BP ≥ 140/90 mmHg, or having a history of hypertension, or currently taking antihypertensive drugs. BMI was classified into groups of < 18.5, 18.5-23.9, 24.0-27.9, and ≥ 28 kg/m^2^. Central obesity was defined as WC ≥ 90 cm for men and ≥ 80 cm for women. The definition of dyslipidemia includes a combination of increased total cholesterol (≥ 200 mg/dL (5.2 mmol/L)), and/or low-density lipoprotein cholesterol (≥ 130 mg/dL (3.4 mmol/L)), and/or triglyceride levels (> 150 mg/dL (1.7 mmol/L)), and/or decreased high-density lipoprotein cholesterol (< 40 mg/dL (1.0 mmol/L)), and/or taking lipid-lowering drugs [19]. Hyperuricemia was detected by the cut-off point of serum uric acid level ≥ 420 *μ*mol/L in males and ≥ 360 *μ*mol/L in females. “Inactive physical exercise” includes “no” or “occasional” (once or twice a week) exercise, and “active physical exercise” was considered “regular” (at least three times a week and each time continue at least 30 minutes). Current cigarette users were regarded as regular smoking (≥ 1 cigarette a day) in the last year, and current drinkers were identified by average alcohol consumption at least 100 mL a day for more than one year.

### 2.6. Statistical Analysis

The characteristics of patients with IFG and diabetes were examined by whether or not the hs-CRP level was reduced. The normally distributed variables, the skewed distributed variables, the classified variables, and the incidence ratio were separately presented as mean ± standard deviation, median (interquartile range), number (proportion), and per 100,000 person-years. Baseline characteristics between groups were compared using the *t*-test or Mann-Whitney *U* for continuous variables depending on the data distribution and *χ*^2^ test for classified variables.

Cox proportional hazard regression models were used to estimate the effect of reduction in hs-CRP levels on kidney outcomes. Model 1 controls for age and sex. Then, BMI (categories), FBG (per 1 mmol/L), dyslipidemia (yes/no), hypertension (yes/no), antihypertensive medicine (yes/no), hyperuricemia (yes/no), cigarette use (current yes/no), alcohol use (current yes/no), exercise (active/inactive), and baseline eGFR (per 1 mL/min/1.73 m^2^) were included into model 2 as potential confounders. For the outcome of kidney function decline, proteinuria (yes/no) was also included in model 2. We conducted a sensitivity analysis by removing participants who had a baseline eGFR < 60 mL/min/1.73 m^2^ in order to avoid the potential impact of reverse causality between the low eGFR and hs-CRP levels. The proportional hazard assumption was checked by the Schoenfeld residual test.

All statistical analyses were conducted using SAS software, version 9.4 (SAS Institute, Cary, NC). Statistical significance was defined as alpha < 0.05 with the two-sided test.

## 3. Results

### 3.1. Baseline Characteristics of Patients

Firstly, there were 3924 patients that were suitable for analysis (Figure 1). As shown in Table 1, the mean age was 54.9 ± 10.8 years, and 76.8% (*N* = 3013) were male. Patients with reduced levels of hs-CRP tended to be younger, were more likely to be male, and have lower levels of BP, BMI, WC, FBG, triglycerides, and baseline hs-CRP and less proportion of proteinuria.

### 3.2. Incidence Rates of Kidney Outcomes

According to the inclusion and exclusion criteria, 3905 patients (55.0 ± 10.8 years, 2998 males) were included into analysis after further removing 19 patients with missing information of eGFR at baseline or subsequent visits for the outcome of kidney function decline. After the mean follow-up of 5.0 ± 1.6 years (19,596 person-years), 398 (10.2%) patients experienced kidney function decline (incidence ratio: 2031/100,000 person-years).

For the outcome of the development of proteinuria, 2049 patients without proteinuria (54.0 ± 10.7 years, 1524 males) at baseline were included into analysis. After the mean follow-up of 5.0 ± 1.6 years (10,242 person-years), 297 (14.5%) patients developed proteinuria (incidence ratio: 2900/100,000 person-years).

For the outcome of the progression of proteinuria, 493 patients with microproteinuria (55.9 ± 10.6 years, 403 males) were included into analysis. After the mean follow-up of 4.8 ± 1.6 years (2370 person-years), 47 (9.5%) patients experienced the progression of proteinuria (incidence ratio: 1983/100,000 person-years).

### 3.3. Effects of Reduction in hs-CRP Levels on Kidney Outcomes

As shown in Table 2, patients with reduction in hs-CRP levels have a lower risk of both kidney function decline (HR 0.73, 95% CI 0.59 to 0.90, and *P* = 0.003) and development of proteinuria (HR 0.72, 95% CI 0.57 to 0.92, and *P* = 0.008), but not progression of proteinuria (HR 0.83, 95% CI 0.43 to 1.60, and *P* = 0.575) after adjusting for age and sex (model 1) as compared to those with no reduction in hs-CRP levels. The results were essentially unchanged after adjusted for potential confounders (model 2).

### 3.4. Sensitivity Analysis

The sensitivity analysis was conducted by rerunning the Cox proportional hazard models after removing patients with eGFR > 60 mL/min/1.73 m^2^. In the fully adjusted models, the results did not change substantially: for kidney function decline (HR 0.69, 95% CI 0.54 to 0.87, and *P* = 0.002) and for development of proteinuria (HR 0.77, 95% CI 0.59 to 1.00, and *P* = 0.053).

## 4. Discussion

Based on a subgroup of patients with IFG or diabetes and high level of hs-CRP from the Kailuan cohort study, we found that reduction in hs-CRP levels was associated with the decreased risk of kidney function decline and development of proteinuria.

Previous studies focused on the effects of high level of hs-CRP at baseline on the kidney outcomes, while whether the reduced hs-CRP level is beneficial to kidney outcomes remains to be evaluated. Our study verified that the reduction in hs-CRP levels was beneficial to kidney outcomes from another perspective. This finding supports the efficacy of treatment targeting at inflammation in improving kidney outcomes.

Nonimmunosuppressive treatment lowering the level of hs-CRP has been shown to be beneficial to kidney outcomes and cardiovascular disease in patients with CKD [20]. A clinical trial including 80 patients showed that CRP lowering with atorvastatin appeared to be effective in eliminating paroxysmal atrial fibrillation in daily life [21]. The Japan Statin Treatment Against Recurrent Stroke trial including 1995 patients with ischemic stroke indicated that reduction in hs-CRP levels by statin treatment was beneficial for recurrent stroke and vascular events within 2 months [22]. Currently, no studies focused on the impact of reduction in hs-CRP levels on kidney disease among diabetic patients with high levels of hs-CRP. There are several studies that supported a positive association between hs-CRP and kidney disease among patients with diabetes and nondiabetes despite remaining controversial [11, 23]. SAVOR-TIMI 53, a trial involving 12,310 patients with type 2 diabetes, found the relationship between increased hs-CRP levels and decline in eGFR or progression of albuminuria [23]. The Finnish Diabetic Nephropathy Study demonstrated that hs-CRP levels are only borderline associated with the progression to a composite kidney outcome (higher albuminuria concentration or ESKD) in 1564 patients with type 1 diabetes after 5.8 years of follow-up [7]. However, the DCCT study did not observe the association between hs-CRP and changes of albuminuria in patients with type 1 diabetes after 9 years of follow-up [11]. Hayashino and colleagues demonstrated that elevated hs-CRP levels could increase the risk of development, not progression of albuminuria in patients with type 2 diabetes after 1 year of follow-up [24]. Instead of ambulatory measurement, these studies used baseline serum hs-CRP which may not properly reveal the long-term inflammatory status as hs-CRP levels show significant variation [25]. The current observational study further enriched the knowledge on their relationship.

Besides statin use, there are several nonimmunosuppressive ways to decrease hs-CRP levels. A systematic review and meta-analysis indicated that the natural soybean protein products may decrease plasma hs-CRP levels as compared with isoflavones from other sources [26]. Regarding exercise, there is plenty of evidence to support that physical exercise reduces inflammatory biomarkers such as CRP in large populations and the magnitude of reduction seems to depend on the cumulative level of physical activity and is sort of akin to the changes by using statins to lower CRP [27]. In addition, a meta-analysis suggested that vitamin D supplementation could benefit to the reduction of circulating hs-CRP levels [28].

Despite the fact that hs-CRP is a practical indictor for risk assessment, many experimental investigations indicated that CRP itself is difficult to be a target for therapy. Following upstream of the inflammatory response from CRP to IL-6 to IL-1, increasing the availability of novel opportunities inhibits the activation of NLRP3 inflammasomes [29]. In addition, a recent study demonstrated that hs-CRP could bind to Fc*γ*RII on apoptotic cells and exacerbate epithelial-mesenchymal transition via the Wnt/*β*-catenin and ERK1/2 signal paths, which could promote the incidence of diabetic kidney disease [30]. However, the potential pathophysiological mechanisms could be complicated and require more research studies to ascertain a target for controlling inflammation in the process of the development and progression of kidney disease.

There were certain limitations in our study. Firstly, the current study was an observational study instead of a randomized controlled trial, so the probability of residual confounding factors cannot be ruled out. Secondly, males account for about 80% of the patients that could limit the applicability of the findings to females. Thirdly, this study is based on employees and relatives of the Kailuan Coal Mine Group, which limits the generalizability of our study. Further studies are needed to validate our results among other populations. Finally, eGFR and proteinuria were tested by using a urine sample, which can lead to the inaccuracy of the classification. Despite these limitations, this study determined that the reduction in hs-CRP levels did favor kidney outcomes.

## 5. Conclusions

In conclusion, reduction in serum hs-CRP levels favors kidney outcomes in patients with IFG or diabetes. However, randomized controlled clinical trials are warranted to further confirm this association.

## Figures and Tables

**Table 1:** Baseline characteristics of patients and divided by reduction in hs-CRP levels.

Characteristics	Total (*n* = 3924)	No reduction in hs-CRP levels (*n* = 2320)	Reduction in hs-CRP levels (*n* = 1604)	*P*
Age (year)	54.9 ± 10.8	56.9 ± 10.4	52.2 ± 10.8	<0.001
Male	3013 (76.8)	1702 (73.4)	1311 (81.7)	<0.001
SBP (mmHg)	137.0 ± 21.6	139.3 ± 21.7	133.8 ± 21.2	<0.001
BMI (kg/m^2^)	26.5 ± 3.7	26.7 ± 3.8	26.1 ± 3.5	<0.001
Waist circumference (cm)	91.6 ± 10.1	93.2 ± 9.8	89.2 ± 9.9	<0.001
FBG (mmol/L)	6.3 (5.9, 7.6)	6.4 (5.9, 7.8)	6.2 (5.8, 7.4)	<0.001
TC (mmol/L)	5.1 (4.5, 5.8)	5.1 (4.5, 5.9)	5.1 (4.4, 5.7)	0.10
TG (mmol/L)	1.6 (1.1, 2.5)	1.7 (1.2, 2.6)	1.5 (1.0, 2.3)	<0.001
LDL-C (mmol/L)	2.3 (1.4, 3.0)	2.1 (0.7, 2.9)	2.5 (2.0, 3.0)	<0.001
HDL-C (mmol/L)	1.5 (1.3, 1.8)	1.5 (1.3, 1.8)	1.5 (1.3, 1.7)	<0.001
Uric acid (*μ*mol/L)	289.6 ± 90.0	285.0 ± 92.4	296.4 ± 85.9	<0.001
eGFR (mL/min/1.73m^2^)	83.6 ± 32.6	84.1 ± 37.7	82.9 ± 23.1	<0.001
Baseline hs-CRP (mg/L)	6.0 (4.0, 9.2)	6.63 (4.4, 9.65)	5.0 (3.7, 8.3)	<0.001
Proteinuria (*N* (%))	681 (18.1)	425 (18.8)	256 (17.3)	<0.001
Cigarette use (*N* (%))	1139 (31.4)	557 (26.9)	582 (37.2)	<0.001
Drinker (*N* (%))	1251 (34.5)	588 (28.5)	663 (42.5)	<0.001
Physical exercise (*N* (%))	615 (17.1)	330 (16.1)	285 (18.3)	0.09
Hypertension (*N* (%))	2295 (58.5)	1472 (63.5)	823 (51.3)	<0.001
Diabetes (*N* (%))	1465 (37.3)	915 (39.4)	550 (34.3)	<0.001
Hypotensive drugs (*N* (%))	692 (34.2)	412 (32.6)	280 (36.8)	0.052

Note: SBP = systolic blood pressure; BMI = body mass index; FBG = fasting blood glucose; TC = total cholesterol; TG = triglyceride; LDL-C = low-density lipoprotein cholesterol; HDL-C = high-density lipoprotein cholesterol; eGFR = estimated glomerular filtration rate.

**Table 2:** Hazard ratios (HRs) and 95% CIs for kidney outcomes.

	No reduction in hs-CRP levels	Reduction in hs-CRP levels
Outcome 1 (*N* = 3905): kidney function decline		
Number of patients	2309	1596
Number of events	259 (11.2%)	139 (8.7%)
Per 100,000 person-years	2308	1659
Model 1, HR (95% CI)	Reference	0.73 (0.59, 0.90)
*P*	—	0.003
Model 2, HR (95% CI)	Reference	0.71 (0.57, 0.89)
*P*	—	0.002
Sensitivity analysis (*N* = 3430), HR (95% CI)	Reference	0.69 (0.54, 0.87)
*P*	—	0.002
Outcome 2 (*N* = 2049): development of proteinuria		
Number of patients	1131	918
Number of events	182 (16.1%)	115 (12.5%)
Per 100,000 person-years	3278	2451
Model 1, HR (95% CI)	Reference	0.72 (0.57, 0.92)
*P*	—	0.008
Model 2, HR (95% CI)	Reference	0.77 (0.61, 0.99)
*P*	—	0.038
Sensitivity analysis (*N* = 1821), HR (95% CI)	Reference	0.77 (0.59, 1.00)
*P*	—	0.053
Outcome 3 (*N* = 493): progression of proteinuria		
Number of patients	328	165
Number of events	33 (10.1%)	14 (8.5%)
Per 100,000 person-years	2154	1672
Model 1, HR (95% CI)	Reference	0.83 (0.43, 1.60)
*P*	—	0.575
Model 2, HR (95% CI)	Reference	0.95 (0.48, 1.88)
*P*	—	0.885

Note: in all three outcomes, model 1 was adjusted for age and sex; model 2 further controlled for BMI, WC, FBG, dyslipidemia, hypertension, antihypertensive drugs, hyperuricemia, cigarette use, drinking, physical exercise, and eGFR. In outcome 1, add proteinuria.

## Data Availability

The datasets used and/or analyzed during the current study are available from the corresponding author on reasonable request.

## References

[1] Alicic R. Z., Rooney M. T., Tuttle K. R. (2017). Diabetic kidney disease: challenges, progress, and possibilities. *Clinical Journal of the American Society of Nephrology*.

[2] Plantinga L. C., Crews D. C., Coresh J. (2010). Prevalence of chronic kidney disease in US adults with undiagnosed diabetes or prediabetes. *Clinical Journal of the American Society of Nephrology*.

[3] (2019). International Diabetes Federation Diabetes Atlas. https://www.diabetesatlas.org/en/sections/demographic-and-geographic-outline.html.

[4] Zhang L., Wang F., Wang L. (2012). Prevalence of chronic kidney disease in China: a cross-sectional survey. *The Lancet*.

[5] Zhang L., Long J., Jiang W. (2016). Trends in chronic kidney disease in China. *The New England Journal of Medicine*.

[6] Li Y., Zhong X., Cheng G. (2017). Hs-CRP and all-cause, cardiovascular, and cancer mortality risk: A meta- analysis. *Atherosclerosis*.

[7] Hansen T. K., on behalf of the FinnDiane Study Group, Forsblom C. (2010). Association between mannose-binding lectin, high-sensitivity C-reactive protein and the progression of diabetic nephropathy in type 1 diabetes. *Diabetologia*.

[8] Kubo S., Kitamura A., Imano H. (2016). Serum albumin and high-sensitivity C-reactive protein are independent risk factors of chronic kidney disease in middle-aged Japanese individuals: the circulatory risk in communities study. *Journal of Atherosclerosis and Thrombosis*.

[9] Aryan Z., Ghajar A., Faghihi-Kashani S., Afarideh M., Nakhjavani M., Esteghamati A. (2018). Baseline high-sensitivity C-reactive protein predicts macrovascular and microvascular complications of type 2 diabetes: a population-based study. *Annals of Nutrition & Metabolism*.

[10] Sarnak M. J., Poindexter A., Wang S. R. (2002). Serum C-reactive protein and leptin as predictors of kidney disease progression in the modification of diet in renal disease study. *Kidney International*.

[11] Lin J., Glynn R. J., Rifai N. (2008). Inflammation and progressive nephropathy in type 1 diabetes in the diabetes control and complications trial. *Diabetes Care*.

[12] Wu S., Huang Z., Yang X. (2012). Prevalence of ideal cardiovascular health and its relationship with the 4-year cardiovascular events in a northern Chinese industrial city. *Circulation: Cardiovascular Quality and Outcomes*.

[13] The Expert Committee on the Diagnosis and Classification of Diabetes Mellitus (2003). Follow-up report on the diagnosis of diabetes mellitus. *Diabetes Care*.

[14] Oda E., Oohara K., Abe A. (2006). The optimal cut-off point of C-reactive protein as an optional component of metabolic syndrome in Japan. *Circulation Journal*.

[15] Levey A. S., Stevens L. A., Schmid C. H. (2009). A new equation to estimate glomerular filtration rate. *Annals of Internal Medicine*.

[16] Lamb E. J., MacKenzie F., Stevens P. E. (2009). How should proteinuria be detected and measured?. *Annals of Clinical Biochemistry*.

[17] Wang A., Liu J., Li C. (2017). Cumulative exposure to high-sensitivity C-reactive protein predicts the risk of cardiovascular disease. *Journal of the American Heart Association*.

[18] Coresh J., Turin T. C., Matsushita K. (2014). Decline in estimated glomerular filtration rate and subsequent risk of end-stage renal disease and mortality. *JAMA*.

[19] Zhu J.-R., Gao R.-L., Zhao S.-P., Lu G.-P., Zhao D., Li J.-J. (2018). 2016 Chinese guidelines for the management of dyslipidemia in adults. *Journal of Geriatric Cardiology*.

[20] Verma A., Ranganna K. M., Reddy R. S., Verma M., Gordon N. F. (2005). Effect of *Rosuvastatin* on C-Reactive Protein and Renal Function in Patients With Chronic Kidney Disease. *The American Journal of Cardiology*.

[21] Dernellis J., Panaretou M. (2005). Effect of C-reactive protein reduction on paroxysmal atrial fibrillation. *American Heart Journal*.

[22] Kitagawa K., Hosomi N., Nagai Y. (2017). Reduction in high-sensitivity C-reactive protein levels in patients with ischemic stroke by statin treatment: hs-CRP sub-study in J-STARS. *Journal of Atherosclerosis and Thrombosis*.

[23] Zelniker T. A., Morrow D. A., Mosenzon O. (2019). Cardiac and inflammatory biomarkers are associated with worsening renal outcomes in patients with type 2 diabetes mellitus: observations from SAVOR-TIMI 53. *Clinical Chemistry*.

[24] Hayashino Y., Mashitani T., Tsujii S., Ishii H., Diabetes Distress and Care Registry at Tenri Study Group (2014). Serum high-sensitivity C-reactive protein levels are associated with high risk of development, not progression, of diabetic nephropathy among Japanese type 2 diabetic patients: a prospective cohort study (Diabetes Distress and Care Registry at Tenri [DDCRT7]). *Diabetes Care*.

[25] Macy E. M., Hayes T. E., Tracy R. P. (1997). Variability in the measurement of C-reactive protein in healthy subjects: implications for reference intervals and epidemiological applications. *Clinical Chemistry*.

[26] Khodarahmi M., Jafarabadi M. A., Moludi J., Abbasalizad Farhangi M. (2019). A systematic review and meta-analysis of the effects of soy on serum hs-CRP. *Clinical Nutrition*.

[27] Plaisance E. P., Grandjean P. W. (2006). Physical activity and high-sensitivity C-reactive protein. *Sports Medicine*.

[28] Chen N., Wan Z., Han S. F., Li B. Y., Zhang Z. L., Qin L. Q. (2014). Effect of vitamin D supplementation on the level of circulating high-sensitivity C-reactive protein: a meta-analysis of randomized controlled trials. *Nutrients*.

[29] Ridker P. M. (2016). From C-reactive protein to interleukin-6 to Interleukin-1. *Circulation Research*.

[30] Zhang L., Shen Z. Y., Wang K. (2019). C-reactive protein exacerbates epithelial-mesenchymal transition through Wnt/*β*-catenin and ERK signaling in streptozocin-induced diabetic nephropathy. *The FASEB Journal*.

